# Echocardiographic Strain Evaluation Shows Persistent Echocardiographic Changes at 1 Year after Diagnosis of Multisystem Inflammatory Syndrome in Children

**DOI:** 10.3390/children11030308

**Published:** 2024-03-05

**Authors:** Jihye You

**Affiliations:** 1 Department of Pediatrics, Children’s Hospital, Jeonbuk National University, Jeonju 54896, Republic of Korea; shilanep@gmail.com; Tel.: +82-63-250-1460; 2 Research Institute of Clinical Medicine of Jeonbuk National University, Jeonju 54907, Republic of Korea

**Keywords:** multisystem inflammatory syndrome in children, COVID-19, echocardiography, strain

## Abstract

Coronavirus disease (COVID-19) is a global pandemic causing multisystem inflammatory syndrome in children (MIS-C). This study evaluated the long-term echocardiographic impact of MIS-C on patients and compared it with that in a healthy control group. Data from 22 children with MIS-C admitted to Jeonbuk National University Hospital and 22 healthy children (control group) were retrospectively analyzed. Echocardiographic data were compared at two distinct time points: diagnosis and 1-year follow-up. At diagnosis, the MIS-C cohort exhibited significantly reduced left ventricular ejection fraction (LVEF), longitudinal strain across the apical 4- and 2-chamber views, and global longitudinal strain (GLS). At 1-year follow-up, the reduced LVEF in the apical 4-chamber, overall longitudinal strain in the apical 4-chamber, and GLS persisted. However, the right ventricular free wall and global strain remained diminished compared with those in the control group. Despite significant changes over time, the LVEF and longitudinal strain in the apical 4-chamber and z-scores of all coronary arteries were normal at baseline and 1-year follow-up. Persistent cardiac alterations were observed in patients with MIS-C, particularly in both ventricular functions. Therefore, middle- to long-term echocardiographic follow-up may help improve understanding and management of long-term echocardiographic implications in patients with post-COVID-19 syndrome.

## 1. Introduction

Coronavirus disease (COVID-19), caused by the severe acute respiratory syndrome coronavirus 2, rapidly became a global pandemic [1] and resulted in multisystem inflammatory syndrome in children (MIS-C). Characterized by a severe inflammatory response affecting multiple organ systems, MIS-C is a rare but critical post-COVID-19 complication observed in pediatric patients [2]. While its precise etiology remains unclear, the disorder is hypothesized to stem from immune dysregulation following infection. This dysregulation is marked by the presence of enduring immunoglobulin G antibodies, elevated capacity for inflammatory monocyte activation, reduced T-cell lymphocyte levels, enhanced CD8+ T-cell activation, and oxidative stress [3,4,5].

MIS-C manifests as a multisystemic condition presenting various symptoms including continuous fever and gastrointestinal disturbances like abdominal discomfort, nausea, and diarrhea. In addition, it involves mucocutaneous manifestations such as eye inflammation, alterations in mucosal tissues, and dermatological signs like skin rashes and limb swellings [6]. Cardiac involvement is observed in 80% of MIS-C cases, varying in clinical presentation and intensive care requirement, overall management, and patient outcomes [7,8]. Treatment primarily involves administering immunoglobulins and aspirin despite a lack of consensus on the optimal immunomodulatory treatment [9,10].

Acute manifestations of MIS-C are well-documented; however, long-term echocardiographic implications remain undetermined. Notably, several studies have reported favorable results with early and medium-term follow-up echocardiographic analyses in patients with MIS-C [11,12,13,14]. Echocardiography, a non-invasive diagnostic tool, offers insights into the cardiac health of patients with MIS-C [12]. Especially, parameters such as the left ventricular (LV) global longitudinal strain (GLS) and right ventricular (RV) strain provide a better understanding of myocardial function and potential damage compared with traditional echocardiographic parameters [15,16,17]. Nevertheless, although several studies have elucidated the midterm outcomes of this condition, the long-term sequelae remain unexplored [18,19,20].

Therefore, this study evaluated the echocardiographic outcomes of patients with MIS-C, focusing on LV GLS, left atrial (LA) strain, and RV strain at 1-year post-diagnosis. Additionally, this study compared these findings with those of a control group to clarify whether the echocardiographic changes observed were specific to MIS-C or a part of normal cardiac variability in children.

## 2. Materials and Methods

### 2.1. Study Design

This retrospective study enrolled all patients aged < 18 years admitted to Jeonbuk National University Children’s Hospital, a tertiary referral center in the Republic of Korea, for MIS-C between 1 January 2021, and 1 October 2022. The MIS-C was diagnosed according to the US Center for Disease Control criteria [21]. The control group was carefully matched with the MIS-C group regarding sex and age, and was comprised of patients who underwent echocardiography primarily because of symptoms such as cardiac murmurs, chest pain, or dyspnea, and had normal heart function. The patients’ demographic data were analyzed. Patients’ clinical data and short-term outcomes have been previously reported [22]. In this investigation, cases necessitating secondary therapeutic measures following initial treatment are classified as ‘refractory’. Refractoriness, particularly in patients first treated with intravenous immunoglobulin, was defined by persistent fever exceeding 36 h after immunoglobulin infusion.

### 2.2. Echocardiography

Echocardiographic examinations were conducted using the Philips IE33 system (Philips Medical Systems, Andover, MA, USA) and General Electric Vivid E95 machines (GE Health Medical, Horten, Norway) before and after 2023, respectively. Echocardiography was performed by a single pediatric cardiologist, J.Y., who recorded two-cycle loop heartbeats for each examination. The choice of the transducer was based on each patient’s body size. Diastolic LV internal diameter (LVIDd) z-scores were calculated following the equations by Pettersen et al. [23]. The control group underwent echocardiography at a single time point to provide a baseline for comparison with the longitudinal data collected from the MIS-C group. All echocardiographic data were analyzed using EchoPAC software, product version 206 (GE Health Medical, Horten, Norway). Early to late diastolic filling velocities ratio (E/A) and early diastolic transmitral flow velocity to early diastolic mitral annular tissue velocity ratio (E/e’), as assessed using pulsed tissue Doppler imaging, were utilized as traditional values to evaluate LV diastolic function. The E/A ratio was manually calculated using the mean value from three beats on the IE33 and Vivid E95 systems. For the E/e’ ratio, the average values from the medial and lateral sides of the mitral valve were employed.

The myocardial tissue was carefully delineated at the point of end-systole on an individual frame, facilitating the calculation of the two-dimensional longitudinal strain (LS) using the echocardiography machine. The LV LS from apical 4-, 3-, and 2-chamber images were analyzed. The ejection fraction (EF) obtained using the Simpson method was automatically measured during the strain measurement. The strain rate during isovolumic relaxation (SR_IVR_) and ratio of early diastolic transmitral flow velocity (E) to the SR_IVR_ were calculated to assess the diastolic function of the heart. LA strain was calculated using apical 4 and 2-chamber views, and RV strain was evaluated using the apical 4-chamber view. The analysis was limited to LV GLS at the initial diagnosis owing to the use of different echocardiographic machines at diagnosis and the 1-year follow-up. However, the LV GLS, LA strain, and RV strain, as well as SR_IVR_ and E/SR_IVR_, could be calculated for the control group using the 1-year follow-up data. The intraobserver coefficient for the apical 4-chamber LS was calculated by re-measuring the value in all enrolled patients using the same methodology. The re-measured values were then compared with those previously measured during the clinical assessment to calculate the intraobserver coefficient.

### 2.3. Statistical Analysis

Statistical analyses were conducted using SPSS Statistics for Windows (version 29.0; IBM Corp., Armonk, NY, USA). Statistical significance was set at *p* < 0.05. Numerical data are presented as median (interquartile range, IQR) and categorical data as frequency (%). To compare the echocardiographic data between the MIS-C and control groups, Mann–Whitney U tests were performed at two time points: diagnosis and 1-year follow-up. These two time points for the MIS-C group were compared with the single time-point data from the echocardiography results of the control group. In addition, Wilcoxon tests were used to compare echocardiographic data within the MIS-C group at diagnosis and 1-year postdiagnosis.

## 3. Results

Table 1 presents the demographic characteristics of the MIS-C and control groups, each comprising 22 participants. The median date when symptoms were first reported in patients diagnosed with MIS-C was July 2022. This period coincided with the prevalence of the Omicron variant of severe acute respiratory syndrome coronavirus-2 in South Korea. The median duration of fever symptoms among these patients was 5.0 days, with an IQR of 4.5 to 5.5 days. Within the cohort of patients with MIS-C, 18.2% required admission to the intensive care unit for monitoring owing to hypotension. However, inotropic support was required in 13.6% of the patients to manage hypotensive conditions. In the study, 22.7% of patients with MIS-C recovered without requiring treatment, whereas the rest received immunoglobulin treatment. In cases where patients presented with hypotension, a combined treatment involving immunoglobulin and steroids was administered as the primary therapy in 29.4% of cases.

Conversely, intravenous immunoglobulin alone was utilized as the initial treatment for patients with stable blood pressure. Overall, steroids were prescribed to 40.9% of patients with MIS-C. Among those who received steroids, 44.4% were undergoing steroid therapy owing to refractory disease. At diagnosis, the median age of participants was 8.0 years (IQR 6.0–10.8) for the MIS-C group and 5.5 years (IQR 5.0–8.8) for the control group, with a *p*-value of 0.326. Baseline echocardiograms, denoted as “time zero” ultrasounds, were performed upon admission during the acute phase of MIS-C. At the 1-year follow-up, the median age in the MIS-C group was 9.0 years (IQR 6.0–13.0). The median interval between the two time points was 393.0 days. The sex distribution was also comparable, with males constituting 45.5% and 54.5% of the MIS-C and control group populations, respectively (*p* = 0.763). Regarding physical growth, the median heights at diagnosis in the MIS-C and control groups were 133.0 (IQR 121.1–155.8) and 117.8 (IQR 110.8–138.3) cm, respectively (*p* = 0.286); at the 1-year follow-up, it increased to 133.4 (IQR 124.5–159.0) cm for the MIS-C group, which did not significantly differ from that of the control group (*p* = 0.148). The median weight at diagnosis in the MIS-C group was 30.5 (IQR 22.8–45.2) kg, compared with 22.2 (IQR 18.8–31.8) kg in the control group (*p* = 0.231); this value increased to 35.4 (IQR 25.0–52.0) kg for the MIS-C group, which did not significantly differ from that of the control group (*p* = 0.084).

### 3.1. Analysis of Echocardiographic Parameters at MIS-C Diagnosis

Echocardiographic evaluation at MIS-C diagnosis revealed some significant differences between the two groups (Table 2). Conventional echocardiographic parameters such as LVIDd z-scores, fractional shortening, EF estimated through the Teichholz formula, LV mass index, tricuspid annular plane systolic excursion (TAPSE), and lateral tricuspid annular peak systolic velocity measured using pulsed tissue Doppler imaging (RVs’) were similar in the two groups. Only the mitral E/e’ ratio showed a median of 10.0 in the MIS-C group, which was higher than the control group’s median of 7.6 (*p* = 0.050).

However, differences were observed between the groups in the LVEF and LS assessed at the apical 4- and 2-chamber views and GLS. The LVEF in the apical 4-chamber view was reduced in the MIS-C group (51.0% with an IQR of 46.0–53.0) compared with that in the control group (58.5% with an IQR of 55.0–60.0) (*p* < 0.001). Similar results were observed in the apical 2-chamber (MIS-C: 53.0% with an IQR of 47.0–60.0, control: 61.0% with an IQR of 59.0–63.8, *p* = 0.010) and total LVEF (MIS-C: 52.0% with an IQR of 47.0–59.0, control: 59.5% with an IQR of 58.2–62.0, *p* = 0.002). Regarding LS, significant differences were observed in the apical 4- and 2-chamber views (*p* < 0.001 and *p* = 0.006, respectively), whereas the apical 3-chamber LS showed no significant difference (*p* = 0.218). Similarly, GLS differed, being lower in the MIS-C group (−17.5% with an IQR of −18.7 to −15.9) compared with the control group (−20.1% with an IQR of −20.9 to −19.1, *p* = 0.001).

### 3.2. Echocardiographic Findings of the 1-Year Follow-Up in Patients with MIS-C

**Figure 1:**
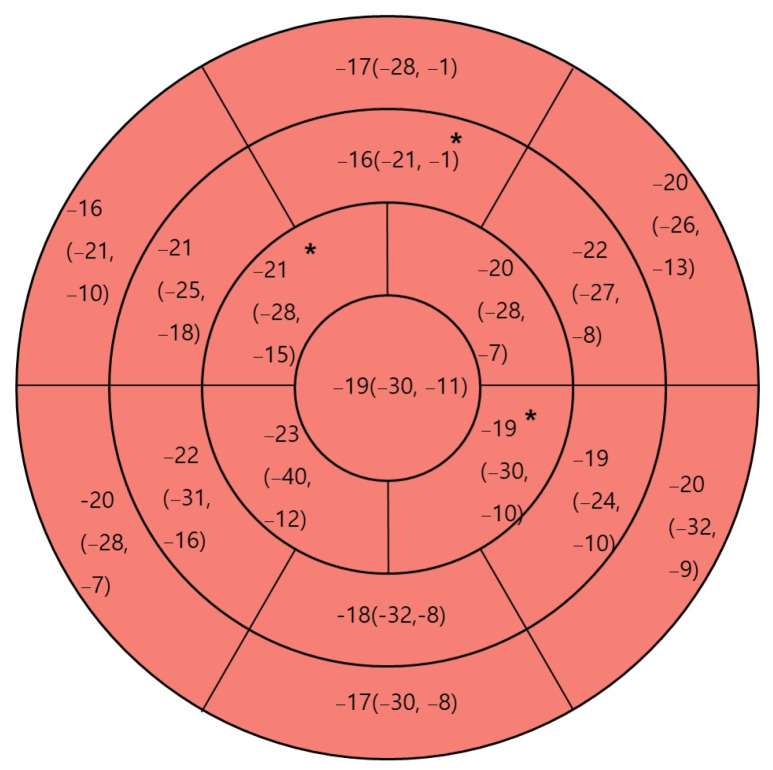
One-year post-diagnosis regional cardiac strain in patients with multisystem inflammatory syndrome in children (MIS-C). Regional strain is depicted as median (minimum, maximum). * *p* < 0.05 compared with control.

**Figure 2:**
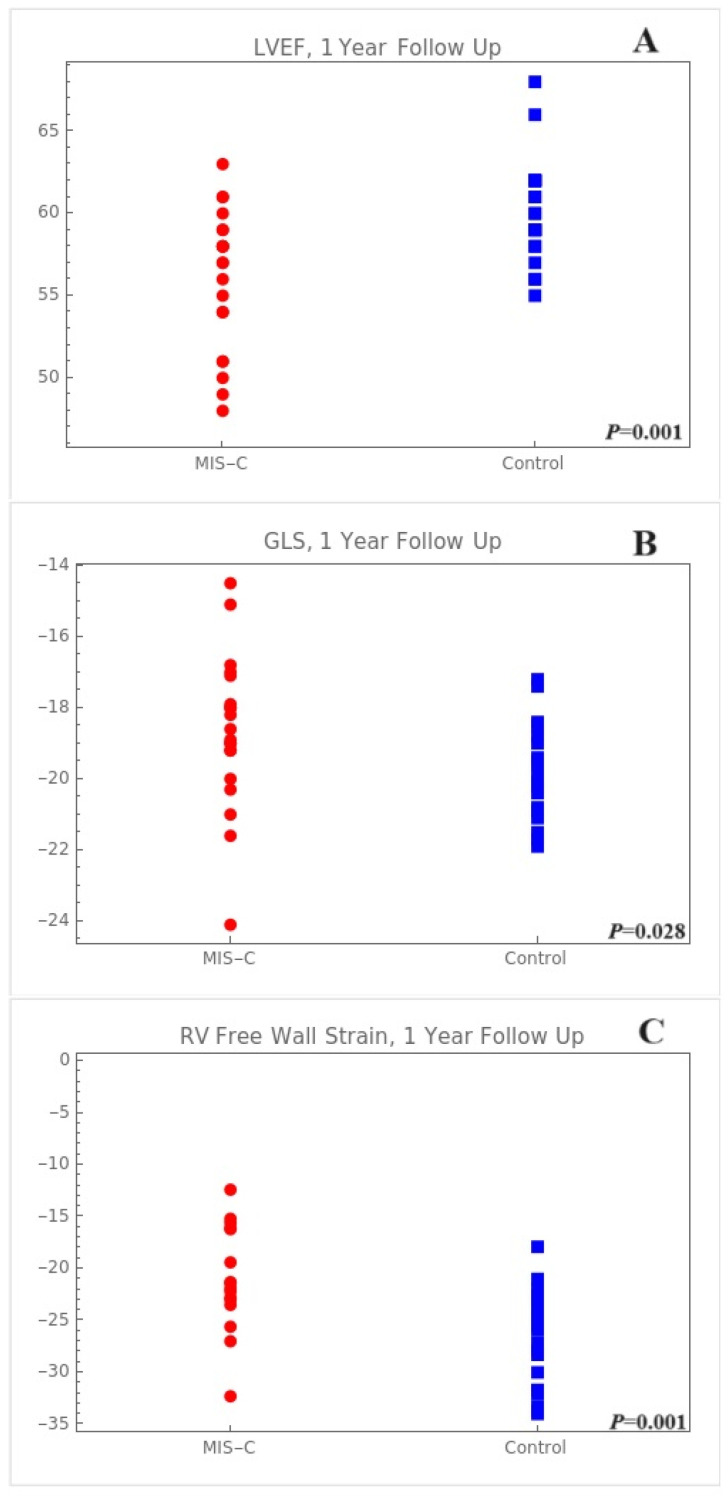
One-year post-diagnosis regional cardiac strain in patients with MIS-C. (**A**) Total LVEF using Simpson method, (**B**) GLS, (**C**) RV free wall. LVEF, left ventricular ejection fraction; GLS, global longitudinal strain; RV, right ventricle.

Echocardiographic evaluations conducted at the 1-year post-diagnosis of MIS-C revealed distinct disparities compared with the control group (Table 3; Figure 1 and Figure 2). Standard echocardiographic measures showed no significant difference between the groups; however, differences were observed in LVEF (Simpson’s 4-chamber and Simpson’s biplane), and in some strain parameters.

The median LVEF measured at the apical 4-chamber view and its IQR was lower in the MIS-C group (54.0%, IQR: 51.0–57.0%) than in the control group (58.5%, IQR: 55.0–60.0%, *p* = 0.003). This trend was also observed in the overall LVEF, where the MIS-C group presented a median value of 57.0% (IQR: 54.0–59.0%) compared with 59.5% (IQR: 58.2–62.0%) in the control group (*p* = 0.002). Regarding LS, a significant difference was observed in the apical 4-chamber view (MIS-C: median of −18.9%, IQR: −20.1% to −17.4% vs. control: median of −20.8%, IQR: −21.9% to −18.9%, *p* = 0.025). However, no significant differences were observed in the apical 3- and 2-chamber views of the LS (*p* = 0.314 and *p* = 0.148, respectively). The LV GLS also showed a significant reduction in the MIS-C group (median: −18.8%, IQR: −19.4% to −17.7%) compared with that in the control group (median: −20.1%, IQR: −20.9% to −19.1%, *p* = 0.015, Figure 1). Compared with the control group, the GLS segmental analysis revealed compromised regional strains in the mid-anterior, apical-anterior, and apical-inferior areas (Figure 1).

Furthermore, the LA strain showed no significant variation between the MIS-C and control groups; however, the RV free wall (median: −20.0%, IQR: −21.7% to −17.5%) and global strains (median: −21.6%, IQR: −23.1% to −16.2%) in the MIS-C cohort at the 1-year follow up were lower as compared to those in the control group (−23.6%, IQR: −25.7% to −22.0% and −25.9%, IQR: −31.7% to −23.5%, respectively), with these differences being statistically significant (*p* = 0.001 and *p* = 0.001, respectively).

### 3.3. Treatment Outcomes in Patients with MIS-C

**Figure 3:**
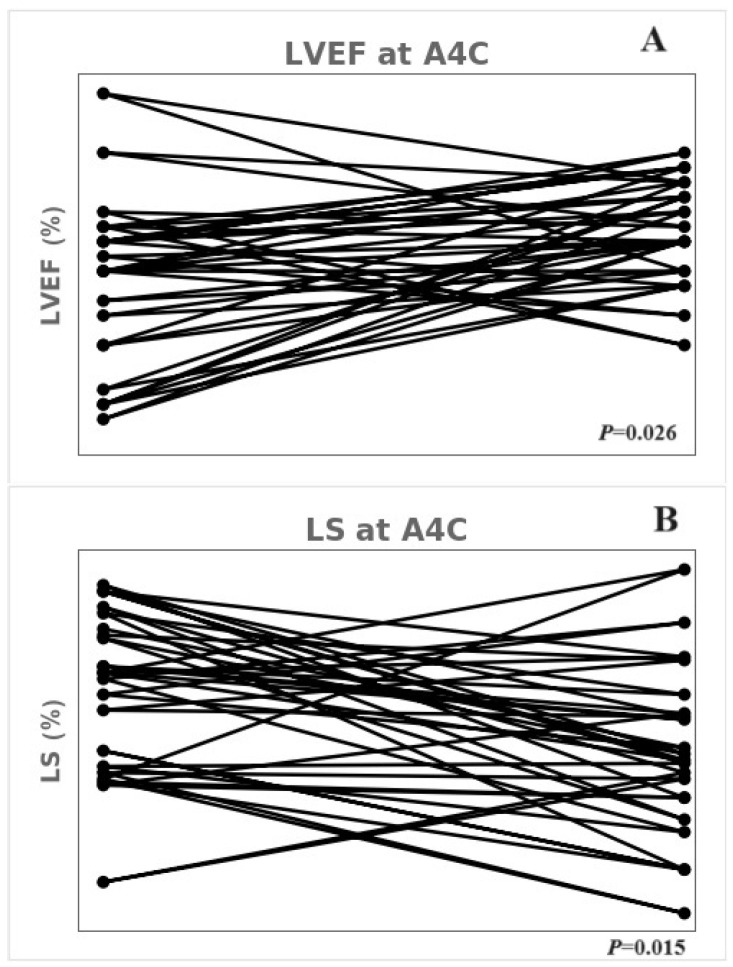
One-to-one corresponding dot plot of LVEF and LS at the apical 4-chamber at diagnosis and after 1 year. (**A**) LVEF using the Simpson method in the apical 4-chamber. (**B**) LS in the apical 4-chamber. LVEF; left ventricular ejection fraction, LS; longitudinal strain.

Some echocardiographic parameters remained reduced at the 1-year follow-up compared with those in the control group; however, significant changes were observed in the LVEF and LS measured in the apical 4-chamber. The E/e’ ratio in the MIS-C group changed from a median of 9.9 (IQR: 7.6–10.7) at diagnosis to 7.9 (IQR: 6.9 to 8.5) at the 1-year follow-up (*p* = 0.005). Despite this reduction, it is worth mentioning that the E/e’ values were within the normal range already at disease presentation, so this is not a normalization but rather a value decrease, while always and consistently being within the normal range [24]. The LVEF in the apical 4-chamber increased from a median of 51.0% (IQR: 46.0–53.2%) at diagnosis to 53.5% (IQR: 51.0–56.2%) at the 1-year follow-up (*p* = 0.030). The absolute LS value in the apical 4-chamber significantly increased from a median of −16.1% (IQR: −19.1% to −14.9%) at diagnosis to −18.9% (IQR: −20.1% to −17.4%) at the 1-year follow-up (*p* = 0.009). Figure 3 shows the one-to-one correspondence dot plot of LVEF and LS in the apical 4-chamber with those at diagnosis and the 1-year follow-up. However, the global LVEF and GLS did not increase significantly at the 1-year follow-up compared with those at diagnosis (Table 4).

Apart from these echocardiographic parameters, z-scores of the coronary arteries were compared between the time of diagnosis and the 1-year follow-up. Significant reductions were observed in the z-scores of all measured coronary arteries. The left main coronary artery (LMCA) z-score significantly decreased from a median of 1.76 (IQR: 1.11–2.35) at diagnosis to −0.04 (IQR: −0.43–0.70) at the 1-year follow-up (*p* < 0.001). Similarly, the left anterior descending artery (LAD), left circumflex artery (LCx), and right coronary artery (RCA) showed significantly decreased z-scores (Table 5). Also here, it is important to underscore that these are all variations within the normal ranges, rather than a normalization since the median z-scores were already within the normal range at diagnosis (Table 5).

### 3.4. Reproducibility Testing

One investigator (J.Y.) performed the echocardiography. The intraobserver coefficient for the apical 4-chamber LS was 0.958.

## 4. Discussion

The findings of this small study suggest that despite some recovery in ventricular function over time, mild subclinical LV and RV dysfunction persisted at 1-year post-diagnosis. This observation suggests a potential interest in ongoing cardiac function follow-up in patients with this condition. Notably, conventional echocardiographic parameters of patients with MIS-C did not differ significantly from those of the controls, indicating that these subclinical ventricular dysfunctions may not have immediate discernible clinical implications. However, the potential long-term effects of these dysfunctions remain unclear.

In children, the normal values for LV GLS vary significantly with age. Consequently, this study compared the patient cohort with a control group of similar age to contextualize these values appropriately. Previous meta-analyses on LV GLS have indicated that the confidence interval for GLS in the age group best represented in the study (2–9 years) is −23.0 to −20.5, and that of the 10–13 years age group is −20.8 to −19.1 [25]. Therefore, the 1-year follow-up value of −18.7 in the MIS-C patient cohort, while approaching normality, was still slightly below these values.

In midterm analyses, several studies focused on segmental LSs in patients with MIS-C, showing that LS recovery is typically primarily observed in LV mid-to-basal areas at 1–2 months post-diagnosis [18,19,20]. Başar et al. highlighted that at 2 months post-diagnosis, the LV apical area in patients with MIS-C showed no significant differences in strain compared with that at the initial diagnosis [20], suggesting a different recovery pattern in that region. In the present study, regional analysis at diagnosis was not feasible because different echocardiographic machines were used to assess LV strain. Consequently, a direct comparison between the findings at diagnosis and 1-year follow-up was impossible. However, when compared with controls, the findings at the 1-year mark revealed that the decreased strain was more pronounced in the apical area than in the commonly implicated basal area. This finding provides a different perspective from those reported by Başar et al., who observed no significant differences in the LV apical area when comparing results at initial diagnosis and the 2-month follow-up in patients with MIS-C [20]. This study’s contrasting results at 1-year highlight the potential for temporal variation in cardiac recovery. Also, this study was conducted in an Asian population, raising the possibility that ethnic differences, among other factors, could have influenced the findings compared with previous studies on regional strain analysis [18,19,20].

Furthermore, the strain in the right ventricle may have implications for the strain in the LV septum. This study did not focus primarily on the cytokine storm aspect; however, the observed correlations in previous research between higher ratios of interleukin-6 and interleukin-8 and global longitudinal strain are noteworthy [26]. This association suggests a nuanced interaction between the immune response and cardiac function. Future research may provide additional insight into the relationship between cytokine profiles and immune responses and the observed mild ventricular dysfunction persisting after 1-year post-diagnosis. This approach could further elucidate the complex interplay between the immune system dynamics and the sustained patterns of cardiac recovery in patients with MIS-C.

A short-term analysis by Matsubara et al. revealed a notable reduction in LA function shortly after diagnosis, with diastolic dysfunction remaining evident at approximately 1-week post-diagnosis [12]. Conversely, the strain differences over time could not be calculated in this study owing to limitations in the baseline assessment of LA function. However, the 1-year post-diagnosis revealed normalized LA function as assessed through strain analysis. Furthermore, despite remaining within the normal range, a significant difference in E/e’ ratio was observed over time in the MIS-C group. This finding indicates the potential LA diastolic function recovery over a longer period, highlighting the dynamic nature of cardiac recovery in patients with MIS-C.

Findings on RV function vary. Consistent with the short-term outcomes reported by Matsubara et al., where RV strain remained compromised [12], the present findings indicate that this reduced RV function persisted even after 1-year post-diagnosis. This persistent reduction in RV strain, particularly when compared with those of controls, indicates the possibility of subclinical echocardiographic effects in patients with MIS-C. The potential for ongoing cardiac attainment exists, as a few reports have indicated limitations in physical exertion following recovery from myocarditis associated with MIS-C [27,28].

The present study showed the absence of involvement of coronary arteries, with normal median z-scores already at diagnosis and, while constantly remaining within normal ranges, even with a reduction in their dimensions over time.

### Limitations

This study provides insights into the long-term echocardiographic outcomes of patients with MIS-C; however, it has some limitations. First, the study’s single-center design limits its generalizability. Second, its retrospective design unavoidably implies some risk of bias. In addition, the modest number of participants limits the statistical robustness and comprehensiveness of the findings. Furthermore, the variation in the echocardiographic equipment used across different time points may have contributed to the discrepancies in our findings. The echocardiography machines used in 2022 differed from those used in 2023. However, all patients with MIS-C were enrolled in 2022, and the 1-year follow-up echocardiographic and control group data were consistently obtained using the same machine.

Most importantly, this was just an echocardiographic study, and additional information such the presence of symptoms, clinical examination findings, observations like heart rate or blood pressure, ECG findings, Holter monitoring, cardiac biomarkers, and exercise test results would be required to evaluate the middle-term cardiac involvement of MIS-C. In addition, while sticking to imaging, cardiac magnetic resonance imaging would provide better insights into total and regional function, volumes, and, particularly, the presence of fibrosis and tissue characterization.

## 5. Conclusions

The findings of this study showed recovery in most echocardiographic parameters among patients with MIS-C; however, other parameters like LVEF obtained using the Simpson method, GLS, and RV strain remained impaired at the 1-year post-diagnosis. These findings suggest the opportunity for further studies to better understand the long-term echocardiographic implications of MIS-C.

## Figures and Tables

**Table 1:** Demographic data of the MIS-C and control groups.

	MIS-C (N = 22)	Control (N = 22)	*p*-Value
Age at diagnosis (year)	8.0 (6.0 to 10.8)	5.5 (5.0 to 8.8)	0.326
Age at 1 year f/u (year)	9.0 (6.0 to 13.0)		0.066
Male (%)	45.5	54.5	0.763
Height (cm)	133.0 (121.1 to 155.8)	117.8 (110.8 to 138.3)	0.286
Height at 1 year f/u (cm)	133.4 (124.5 to 159.0)		0.148
Weight (kg)	30.5 (22.8 to 45.2)	22.2 (18.8 to 31.8)	0.231
Weight at 1 year f/u (kg)	35.4 (25.0 to 52.0)		0.084
Organ involvement (N, %)			
Cardiac involvement	21 (95.5)		
Troponin-T (ng/mL)			
N-terminal prohormone of brain natriuretic peptide (pg/mL)			
Renal involvement	7 (31.8)		
Respiratory involvement	4 (18.2)		
Hematologic involvement	18 (81.8)		
Gastrointestinal involvement	19 (86.4)		
Neurologic involvement	6 (27.3)		

MIS-C, multisystem inflammatory syndrome in children.

**Table 2:** Echocardiographic parameters at MIS-C diagnosis compared with those in the control group.

	MIS-C	Control	*p*-Value
LVIDd z score	−0.1 (−0.3 to 0.6)	−0.0 (−0.6 to 0.2)	0.164
FS (%)	36.5 (30.8 to 40.0)	38.2 (33.6 to 40.2)	0.336
EF (%)	67.2 (59.7 to 71.1)	66.4 (62.6 to 71.7)	0.656
LVMI (g/m^2^)	72.2 (69.2 to 78.5)	70.8 (58.2 to 76.0)	0.330
TAPSE (mm)	18.4 (16.7 to 22.5)	20.1 (18.2 to 21.5)	0.627
RV s’ (cm/s)	12.0 (10.2 to 12.8)	12.0 (11.0 to 13.0)	0.380
Mitral E/A	1.6 (1.4 to 1.8)	1.7 (1.6 to 1.8)	0.211
Mitral E/e’	10.0 (7.7 to 10.7)	7.6 (6.6 to 8.5)	0.050
LVEF (apical 4-chamber, %)	51.0 (46.0 to 53.0)	58.5 (55.0 to 60.0)	<0.001
LVEF (apical 2-chamber, %)	53.0 (47.0 to 60.0)	61.0 (59.0 to 63.8)	0.010
LVEF (total, %)	52.0 (47.0 to 59.0)	59.5 (58.2 to 62.0)	0.002
LS at apical 4-chamber (%)	−16.1 (−19.0 to −14.8)	−20.8 (−21.9 to −18.9)	<0.001
LS at apical 3-chamber (%)	−17.3 (−18.3 to −14.1)	−17.7 (−20.2 to −16.4)	0.218
LS at apical 2-chamber (%)	−18.4 (−20.1 to −16.9)	−21.1 (−22.7 to −19.8)	0.006
GLS (%)	−17.5 (−18.7 to −15.9)	−20.1 (−20.9 to −19.1)	0.001

MIS-C, multisystem inflammatory syndrome in children; LVIDd, left ventricular internal dimension at end-diastole; FS, fractional shortening; EF, ejection fraction; LVMI, left ventricular mass index; TAPSE, tricuspid annular plane systolic excursion; RV s’, lateral tricuspid annular peak systolic velocity by pulsed tissue Doppler imaging; E/A, early to late diastolic filling velocities ratio; E/e’, early diastolic transmitral flow velocity to early diastolic mitral annular tissue velocity by pulsed tissue Doppler imaging; LVEF, left ventricular ejection fraction by the Sympson method; LS, longitudinal strain; GLS, global longitudinal strain.

**Table 3:** Echocardiographic parameters in the MIS-C group compared with those in the control group at the 1-year follow-up.

	MIS-C	Control	*p*-Value
LVIDd z score	−0.2 (−0.7 to 0.3)	−0.0 (−0.6 to 0.2)	0.952
FS (%)	36.6 (33.6 to 38.8)	38.2 (33.6 to 40.2)	0.535
EF (%)	68.9 (63.0 to 70.9)	66.4 (62.6 to 71.7)	0.913
LVMI (g/m^2^)	63.2 (56.3 to 72.6)	70.8 (58.2 to 76.0)	0.239
TAPSE (mm)	20.1 (18.0 to 21.7)	20.1 (18.2 to 21.5)	0.743
RV s’ (cm/s)	12.0 (10.0 to 12.0)	12.0 (11.0 to 13.0)	0.154
Mitral E/A	1.7 (1.5 to 1.9)	1.7 (1.6 to 1.8)	0.660
Mitral E/e’	7.7 (6.7 to 8.4)	7.6 (6.6 to 8.5)	0.989
LVEF (apical 4-chamber, %)	54.0 (51.0 to 57.0)	58.5 (55.0 to 60.0)	0.003
LVEF (apical 2-chamber, %)	59.0 (56.0 to 64.0)	61.0 (59.0 to 63.8)	0.124
LVEF (total, %)	57.0 (54.0 to 59.0)	59.5 (58.2 to 62.0)	0.002
LS at apical 4-chamber (%)	−18.9 (−20.1 to −17.4)	−20.8 (−21.9 to −18.9)	0.025
LS at apical 3-chamber (%)	−17.3 (−18.5 to −15.1)	−17.7 (−20.2 to −16.4)	0.314
LS at apical 2-chamber (%)	−19.9 (−21.3 to −17.8)	−21.0 (−22.7 to −19.8)	0.148
GLS (%)	−18.8 (−19.4 to −17.7)	−20.1 (−20.9 to −19.1)	0.015
SR_IVR_ (s^−1^)	0.3 (0.2 to 0.3)	0.2 (0.2 to 0.3)	0.300
E/SR_IVR_ (cm)	389.7 (335.2 to 450.0)	420.6 (340.5 to 466.0)	0.375
4-chamber reservoir strain (%)	38.0 (35.0 to 40.0)	38.0 (34.5 to 41.8)	1.000
2-chamber reservoir strain (%)	40.0 (33.0 to 43.0)	43.0 (36.2 to 47.5)	0.202
Global LA reservoir strain (%)	37.0 (35.0 to 43.0)	40.0 (36.0 to 43.8)	0.551
RV free wall strain (%)	−20.0 (−21.7 to −17.5)	−23.6 (−25.7 to −22.0)	0.001
RV global strain (%)	−21.6 (−23.1 to −16.2)	−25.9 (−31.7 to −23.5)	0.001

MIS-C, multisystem inflammatory syndrome in children; LVIDd, left ventricular internal dimension at end-diastole; FS, fractional shortening; EF, ejection fraction; LVMI, left ventricular mass index; TAPSE, tricuspid annular plane systolic excursion; RV s’, lateral tricuspid annular peak systolic velocity by pulsed tissue Doppler imaging; E/A, early to late diastolic filling velocities ratio; E/e’, early diastolic transmitral flow velocity to early diastolic mitral annular tissue velocity by pulsed tissue Doppler imaging; LVEF, left ventricular ejection fraction by Sympson method; LS, longitudinal strain; GLS, global longitudinal strain; SR_IVR_, strain rate during isovolumic relaxation time; E/SR_IVR_, ratio of early diastolic transmitral flow velocity (E) to the SR_IVR_; LA, left atrium; RV, right ventricle.

**Table 4:** Comparison of echocardiographic findings at diagnosis and 1-year follow-up.

	At Diagnosis	1-Year Follow-Up	*p*-Value
LVIDd z score	−0.2 (−0.3 to 0.6)	−0.2 (−0.7 to 0.3)	0.111
FS (%)	36.4 (30.7 to 40.0)	36.6 (33.6 to 38.8	0.609
EF (%)	66.8 (59.6 to 71.2)	68.9 (63.0 to 70.9)	0.191
LVMI (g/m^2^)	72.2 (69.2 to 78.5	63.2 (55.9 to 74.5)	0.278
TAPSE (mm)	18.2 (16.4 to 22.9)	20.1 (18.0 to 21.7)	0.475
RV s’ (cm/s)	12.0 (10.0 to 13.0)	11.0 (10.0 to 12.0)	0.427
Mitral E/A	1.6 (1.4 to 1.8)	1.66 (1.5 to 1.9)	0.050
Mitral E/e’	9.9 (7.6 to 10.7)	7.9 (6.9 to 8.5)	0.005
LVEF (apical 4-chamber, %)	51.0 (46.0 to 53.2)	53.5 (51.0 to 56.2)	0.030
LVEF (apical 2-chamber, %)	53.5 (47.75 to 61.5)	59.0 (54.5 to 61.0)	0.298
LVEF (total, %)	52.0 (47.0 to 59.2)	56.5 (53.25 to 58.2)	0.175
LS at apical 4-chamber (%)	−16.1 (−19.1 to −14.9)	−18.9 (−20.1 to −17.4)	0.009
LS at apical 3-chamber (%)	−17.3 (−18.25 to −14.5)	−16.9 (−18.45 to −14.7)	0.978
LS at apical 2-chamber (%)	−18.4 (−20.1 to −17.0)	−19.7 (−20.9 to −17.6)	0.860
GLS (%)	−17.5 (−19.0 to −16.2)	−18.6 (−19.1 to −17.6)	0.330

MIS-C, multisystem inflammatory syndrome in children; LVIDd, left ventricular internal dimension at end-diastole; FS, fractional shortening; EF, ejection fraction; LVMI, left ventricular mass index; TAPSE, tricuspid annular plane systolic excursion; RV s’, lateral tricuspid annular peak systolic velocity by pulsed tissue Doppler imaging; E/A, early to late diastolic filling velocities ratio; E/e’, early diastolic transmitral flow velocity to early diastolic mitral annular tissue velocity by pulsed tissue Doppler imaging; LVEF, left ventricular ejection fraction by the Sympson method; LS, longitudinal strain; GLS, global longitudinal strain.

**Table 5:** Comparison of the coronary artery z-scores at diagnosis and 1-year follow-up.

	At Diagnosis	1-Year Follow-Up	*p*-Value
LMCA	1.76 (1.11 to 2.35)	−0.04 (−0.43 to 0.70)	<0.001
LAD	1.46 (0.34 to 1.96)	0.14 (−0.36 to 0.89)	<0.001
LCx	0.76 (0.05 to 1.70)	−0.27 (−0.79 to 0.35)	0.004
RCA	0.80 (0.17 to 1.29)	−0.08 (−0.49 to 0.16)	<0.001

LMCA, left main coronary artery; LAD, left anterior descending coronary artery; LCx, left circumflex coronary artery; RCA, right coronary artery.

## Data Availability

The data presented in this study are available on request from the corresponding author. The data are not publicly available due to the research data are confidential.

## References

[1] Sharma A., Tiwari S., Deb M.K., Marty J.L. (2020). Severe acute respiratory syndrome coronavirus-2 (SARS-CoV-2): A global pandemic and treatment strategies. Int. J. Antimicrob. Agents.

[2] Rafferty M.S., Burrows H., Joseph J.P., Leveille J., Nihtianova S., Amirian E.S. (2021). Multisystem inflammatory syndrome in children (MIS-C) and the coronavirus pandemic: Current knowledge and implications for public health. J. Infect. Public Health.

[3] Lee P.Y., Day-Lewis M., Henderson L.A., Friedman K.G., Lo J., Roberts J.E., Lo M.S., Platt C.D., Chou J., Hoyt K.J. (2020). Distinct clinical and immunological features of SARS-CoV-2-induced multisystem inflammatory syndrome in children. J. Clin. Investig..

[4] Feldstein L.R., Rose E.B., Horwitz S.M., Collins J.P., Newhams M.M., Son M.B.F., Newburger J.W., Kleinman L.C., Heidemann S.M., Martin A.A. (2020). Multisystem inflammatory syndrome in U.S. Children and adolescents. N. Engl. J. Med..

[5] Perrone S., Cannavò L., Manti S., Rullo I., Buonocore G., Esposito S.M.R., Gitto E. (2022). Pediatric multisystem syndrome associated with SARS-CoV-2 (MIS-C): The interplay of oxidative stress and inflammation. Int. J. Mol. Sci..

[6] Nakra N.A., Blumberg D.A., Herrera-Guerra A., Lakshminrusimha S. (2020). Multi-system inflammatory syndrome in children (MIS-C) following SARS-CoV-2 infection: Review of clinical presentation, hypothetical pathogenesis, and proposed management. Children.

[7] Wu E.Y., Campbell M.J. (2021). Cardiac manifestations of multisystem inflammatory syndrome in children (MIS-C) following COVID-19. Curr. Cardiol. Rep..

[8] Alsaied T., Tremoulet A.H., Burns J.C., Saidi A., Dionne A., Lang S.M., Newburger J.W., de Ferranti S., Friedman K.G. (2021). Review of cardiac involvement in multisystem inflammatory syndrome in children. Circulation.

[9] Henderson L.A., Canna S.W., Friedman K.G., Gorelik M., Lapidus S.K., Bassiri H., Behrens E.M., Ferris A., Kernan K.F., Schulert G.S. (2020). American College of Rheumatology clinical guidance for multisystem inflammatory syndrome in children associated with SARS-CoV-2 and hyperinflammation in pediatric COVID-19. version 1. Arthritis Rheumatol..

[10] Mahmoud S., El-Kalliny M., Kotby A., El-Ganzoury M., Fouda E., Ibrahim H. (2022). Treatment of MIS-C in children and adolescents. Curr. Pediatr. Rep..

[11] Garbin M., Raso I., Piersanti A., Gianolio L., De Silvestri A., Calcaterra V., Corti C.G., Nespoli L.F., Santacesaria S., Fini G. (2022). Advanced echocardiographic analysis in medium-term follow-up of children with previous multisystem inflammatory syndrome. Children.

[12] Matsubara D., Kauffman H.L., Wang Y., Calderon-Anyosa R., Nadaraj S., Elias M.D., White T.J., Torowicz D.L., Yubbu P., Giglia T.M. (2020). Echocardiographic findings in pediatric multisystem inflammatory syndrome associated with COVID-19 in the United States. J. Am. Coll. Cardiol..

[13] Fremed M.A., Farooqi K.M. (2022). Longitudinal outcomes and monitoring of patients with multisystem inflammatory syndrome in children. Front. Pediatr..

[14] Capone C.A., Misra N., Ganigara M., Epstein S., Rajan S., Acharya S.S., Hayes D.A., Kearney M.B., Romano A., Friedman R.A. (2021). Six month follow-up of patients with multi-system inflammatory syndrome in children. Pediatrics.

[15] Collier P., Phelan D., Klein A. (2017). A test in context: Myocardial strain measured by speckle-tracking echocardiography. J. Am. Coll. Cardiol..

[16] Mouton S., Ridon H., Fertin M., Pentiah A.D., Goémine C., Petyt G., Lamblin N., Coisne A., Foucher-Hossein C., Montaigne D. (2017). 2D-speckle tracking right ventricular strain to assess right ventricular systolic function in systolic heart failure. Analysis of the right ventricular free and posterolateral walls. Int. J. Cardiol..

[17] Di Nardo M., Franceschini A., Tissieres P., Chinali M. (2022). What is new on paediatric echocardiography for the diagnosis, management and follow-up of the multisystem inflammatory syndrome associated with COVID-19?. Children.

[18] Wong J., Theocharis P., Regan W., Pushparajah K., Stephenson N., Pascall E., Cleary A., O’Byrne L., Savis A., Miller O. (2022). Medium-term cardiac outcomes in young people with multi-system inflammatory syndrome: The era of COVID-19. Pediatr. Cardiol..

[19] He M., Leone D.M., Frye R., Ferdman D.J., Shabanova V., Kosiv K.A., Sugeng L., Faherty E., Karnik R. (2022). Longitudinal assessment of global and regional left ventricular strain in patients with multisystem inflammatory syndrome in children (MIS-C). Pediatr. Cardiol..

[20] Başar E.Z., Usta E., Akgün G., Güngör H.S., Sönmez H.E., Babaoğlu K. (2022). Is strain echocardiography a more sensitive indicator of myocardial involvement in patients with multisystem inflammatory syndrome in children (MIS-C) associated with SARS-CoV-2?. Cardiol. Young.

[21] Melgar M., Lee E.H., Miller A.D., Lim S., Brown C.M., Yousaf A.R., Zambrano L.D., Belay E.D., Godfred-Cato S., Abrams J.Y. (2022). Council of State and Territorial Epidemiologists/CDC Surveillance Case Definition for Multisystem Inflammatory Syndrome in Children Associated with SARS-CoV-2 Infection—United States. MMWR Recomm. Rep..

[22] You S.D., Kim J.H., You J. (2023). Clinical characteristics and short-term outcomes of multisystem inflammatory syndrome in a country with a high prevalence of KD. Front. Pediatr..

[23] Pettersen M.D., Du W., Skeens M.E., Humes R.A. (2008). Regression equations for calculation of z scores of cardiac structures in a large cohort of healthy infants, children, and adolescents: An echocardiographic study. J. Am. Soc. Echocardiogr..

[24] Choi S.H., Eun L.Y., Kim N.K., Jung J.W., Choi J.Y. (2016). Myocardial tissue doppler velocity in child growth. J. Cardiovasc. Ultrasound.

[25] Levy P.T., Machefsky A., Sanchez A.A., Patel M.D., Rogal S., Fowler S., Yaeger L., Hardi A., Holland M.R., Hamvas A. (2016). Reference ranges of left ventricular strain measures by two-dimensional speckle-tracking echocardiography in children: A systematic review and meta-analysis. J. Am. Soc. Echocardiogr..

[26] Chang J.C., Matsubara D., Morgan R.W., Diorio C., Nadaraj S., Teachey D.T., Bassiri H., Behrens E.M., Banerjee A. (2021). Skewed cytokine responses rather than the magnitude of the cytokine storm may drive cardiac dysfunction in multisystem inflammatory syndrome in children. J. Am. Heart Assoc..

[27] McAree D., Griffith G.J., Husain N., Koenig P., Carr M., Ward K. (2023). Multisystem inflammatory syndrome in children (MIS-C): Reduced exercise duration and capacity at six month follow-up. Pediatr. Cardiol..

[28] Mainzer G., Zucker-Toledano M., Hanna M., Bar-Yoseph R., Kodesh E. (2023). Significant exercise limitations after recovery from MIS-C related myocarditis. World J. Pediatr..

